# Major Complex and Minimally Invasive Cancer Surgery Can Be Delivered Safely During the COVID-19 Pandemic

**DOI:** 10.1245/s10434-021-09888-x

**Published:** 2021-04-01

**Authors:** Bilal Alkhaffaf, Mohamed Alasmar

**Affiliations:** 1 Department of Oesophago-Gastric Surgery, Salford Royal Foundation Trust, Salford Royal Hospital, Manchester, UK; 2 Division of Cancer Sciences, School of Medical Sciences, Faculty of Biology, Medicine and Health, University of Manchester, Manchester, UK

## Past

Serious questions have arisen surrounding the safety of undertaking major complex (cancer) surgery during the current pandemic. Are patients at significant risk of acquiring COVID-19, which may lead to devastating outcomes in the perioperative period?^1^ Are health care workers at risk from patients during long procedures or potential aerosolization from minimally invasive surgery (MIS)?^2^ What precautions are necessary to reduce these potential risks? Should surgical programs continue if resources needed to rescue patients from life-threatening complications are being diverted to deal with surges in COVID-19 cases? These are questions that have not been answered with clear direction, resulting in heightened anxiety among patients, many of whom have consequently avoided non–COVID-related medical care.^3^ To begin addressing these concerns, the current study examined the precautions taken by health care services and the outcomes of major complex surgery in the field of esophageal and gastric cancer.

## Present

A prospective multi-center study was undertaken in nine high-volume European surgical centers serving regions heavily affected by COVID-19.^4^ Precautions to mitigate the risks of COVID-19 varied between the centers, although as a minimum, patients were swab-tested preoperatively and managed on “cold” pathways and in areas of the hospital that had no management of COVID-19-positive patients. Esophageal and gastric resection surgeries (57 % MIS) were safely undertaken in 158 cases, with no patients acquiring COVID-19 during the perioperative period. Low levels of morbidity and mortality were achieved, suggesting that the facilities were able to safely manage potentially life-threatening complications. Levels of personal protective equipment in the operating room varied for health care workers between centers. Of the 403 health care workers involved in the operative care of patients, 313 (78 %) completed a COVID-19 health questionnaire. Only two of the health care workers tested positive for COVID-19 during the study period, with the source identified as non-hospital acquisition in both cases. The results suggest that despite significant population levels of COVID-19 and in the context of adequate precautions and hospital resources, major complex surgery can be safely undertaken without significant risk to patients or staff.

## Future

Updated guidance is required concerning minimum precautions that should be exercised to facilitate the safe continuation of complex major surgical programs. Prospective and transparent reporting of all perioperative outcomes is required by other groups to provide continued reassurance that our findings are generalizable in other regions and in differing periods during the pandemic. Reassurance must be provided to patients and their carers that complex surgical management is being safely delivered and that they should not avoid attending the hospital for life-saving treatment.

## References

[1] Nepogodiev D, Bhangu A, Glasbey JC (2020). Mortality and pulmonary complications in patients undergoing surgery with perioperative SARS-CoV-2 infection: an international cohort study. Lancet..

[2] Pavan N, Crestani A, Abrate A (2020). Risk of virus contamination through surgical smoke during minimally invasive surgery: a systematic review of the literature on a neglected issue revived in the COVID-19 pandemic era. Eur Urol Focus..

[3] Czeisler MÉ, Marynak K, Clarke KEN (2020). Delay or avoidance of medical care because of COVID-19-related concerns–United States, June 2020. MMWR Morb Mortal Wkly Rep..

[4] Alasmar M, Kausar A, Borgstein AB-J, et al. Is re-introducing major open and minimally invasive surgery during COVID-19 safe for patients and healthcare workers? An international, multi-center cohort study in the field of Oesophago-gastric surgery. *Ann Surg Oncol*. 10.1245/s10434-021-09885-0. 10.1245/s10434-021-09885-0 PMC8053024 33866473

